# Nuclear Trafficking of the Rabies Virus Interferon Antagonist P-Protein Is Regulated by an Importin-Binding Nuclear Localization Sequence in the C-Terminal Domain

**DOI:** 10.1371/journal.pone.0150477

**Published:** 2016-03-03

**Authors:** Caitlin L. Rowe, Kylie M. Wagstaff, Sibil Oksayan, Dominic J. Glover, David A. Jans, Gregory W. Moseley

**Affiliations:** 1 Viral Pathogenesis Laboratory, Department of Biochemistry and Molecular Biology, Bio21 Molecular Science and Biotechnology Institute, The University of Melbourne, Parkville, Victoria, Australia; 2 Nuclear Signaling Laboratory, Department of Biochemistry and Molecular Biology, Monash University, Clayton, Victoria, Australia; The Hong Kong Polytechnic University, HONG KONG

## Abstract

Rabies virus P-protein is expressed as five isoforms (P1-P5) which undergo nucleocytoplasmic trafficking important to roles in immune evasion. Although nuclear import of P3 is known to be mediated by an importin (IMP)-recognised nuclear localization sequence in the N-terminal region (N-NLS), the mechanisms underlying nuclear import of other P isoforms in which the N-NLS is inactive or has been deleted have remained unresolved. Based on the previous observation that mutation of basic residues K214/R260 of the P-protein C-terminal domain (P-CTD) can result in nuclear exclusion of P3, we used live cell imaging, protein interaction analysis and *in vitro* nuclear transport assays to examine in detail the nuclear trafficking properties of this domain. We find that the effect of mutation of K214/R260 on P3 is largely dependent on nuclear export, suggesting that nuclear exclusion of mutated P3 involves the P-CTD-localized nuclear export sequence (C-NES). However, assays using cells in which nuclear export is pharmacologically inhibited indicate that these mutations significantly inhibit P3 nuclear accumulation and, importantly, prevent nuclear accumulation of P1, suggestive of effects on NLS-mediated import activity in these isoforms. Consistent with this, molecular binding and transport assays indicate that the P-CTD mediates IMPα2/IMPβ1-dependent nuclear import by conferring direct binding to the IMPα2/IMPβ1 heterodimer, as well as to a truncated form of IMPα2 lacking the IMPβ-binding autoinhibitory domain (ΔIBB-IMPα2), and IMPβ1 alone. These properties are all dependent on K214 and R260. This provides the first evidence that P-CTD contains a genuine IMP-binding NLS, and establishes the mechanism by which P-protein isoforms other than P3 can be imported to the nucleus. These data underpin a refined model for P-protein trafficking that involves the concerted action of multiple NESs and IMP-binding NLSs, and highlight the intricate regulation of P-protein subcellular localization, consistent with important roles in infection.

## Introduction

The majority of molecular transport between the cytoplasm and nucleus of eukaryotic cells takes place through nuclear pore complexes (NPCs), which are composed of nucleoporin proteins embedded in the otherwise impermeable nuclear envelope. Active translocation of proteins occurs *via* a highly organized signal-dependent process whereby nuclear localization (NLS) and/or nuclear export (NES) sequences within a cargo protein mediate interaction with cellular nuclear import and export receptors (importins (IMPs) and exportins (EXPs), respectively). IMP/EXP interaction with nucleoporins then effects transport through the NPC [1].

NLSs are generally short modular monopartite sequences encompassing a single stretch of basic residues, as in the SV40 large T-antigen NLS (T-ag NLS: PKKKRKV) [2], or bipartite sequences encompassing two basic residue-rich sequences separated by a linker region, as in nucleoplasmin (KR-10 residue linker-KKKK) [3]. Conformational NLSs have also been described, which appear to be dependent on the domain structure of the cargo protein and so cannot function when expressed out of context [4]. In classical nuclear import pathways, the NLS is recognized by a member of the IMPα family in a complex with a member of the IMPβ family. In this context, binding of IMPβ to the IMPβ-binding domain (IBB) of IMPα relieves the auto-inhibitory effect of IBB, enabling interaction of IMPα2 with the NLS [1]; IMPβ also mediates interaction with nucleoporins to translocate the cargo protein-IMP complex through the NPC and into the nucleus [1]. For some cargoes, the NLS interacts directly with IMPβ for transport without the requirement for IMPα [5–7]. Nuclear export occurs in an analogous fashion, with the NES recognized by a member of the EXP family, of which CRM1 is the best characterized, and interactions of the EXP with the NPC mediating transport to the cytoplasm [8].

The host cell nuclear trafficking machinery is commonly exploited by viruses with nuclear replication cycles to mediate genome delivery and export. However, many viruses with cytoplasmic life cycles, such as the lyssaviruses (a genus of lethal zoonotic viruses that includes rabies virus (RABV) and Australian bat lyssavirus) and paramyxoviruses, also encode proteins that target the nucleus, including RABV phosphoprotein (P-protein). This appears to enable viral interference with nuclear functions including host gene transcription and signaling involved in innate antiviral immunity [9–12].

**Fig 1:**
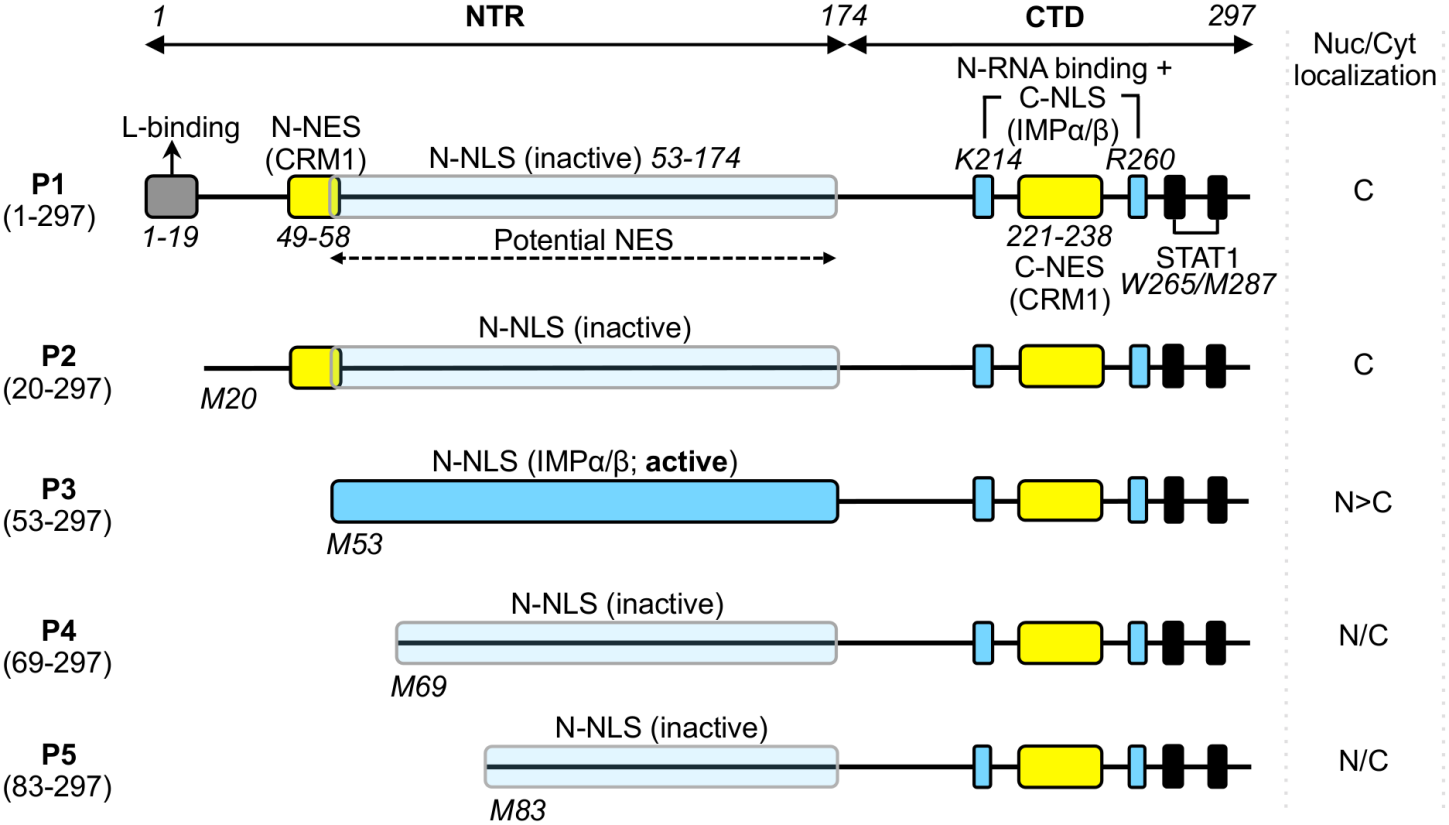
Domain structure of RABV P-protein. P-protein is shown schematically with key domains/sequences indicated; residue positions are indicated by italicized numbering. The RABV P gene encodes full length P1 (residues 1–297) and N-terminally truncated isoforms P2-P5 (expressed *via* ribosomal leaky scanning that initiates translation from internal in-frame AUG codons corresponding to methionines M20, M53, M69 and M83 of P1 [13]). P1 alone contains residues 1–19 that are required for association with the viral L-protein so that P1 can act as the polymerase cofactor [18]. All P-protein isoforms contain the CTD which incorporates the binding sites for viral genome–associated N-protein (N-RNA), [17,23–25] and STAT1 (black boxes) [11,19,26]. P-protein interaction with EXPs and IMPs is mediated through two CRM-1-binding NESs (yellow boxes) [27,28] and two IMPα/β-binding NLSs (blue boxes, described elsewhere [9,27] and in this study), located in both the NTR and CTD. A third potential NES has been suggested to exist within NTR residues 53–174 [9]. The NTR-localized N-NLS is activated in P3 due to truncation of residues 1–52, which also truncates/deactivates the N-NES [9,27]. The P-CTD localized C-NLS, which includes key residues K214/R260 (blue boxes) of a positively charged patch on the surface of the CTD, is characterized in this study; the positive patch has also been implicated in binding to N-RNA [19,24,29–31], suggesting that the N-RNA-binding site overlaps with the C-NLS [24,25,27,28]. The nucleocytoplasmic (Nuc/Cyt) localisation of each isoform is indicated (cytoplasmic (C), nuclear (N), diffuse (N/C)).

RABV P-protein is expressed as full length (P1) protein, and as four N-terminally truncated isoforms (P2-P5), which are generated in infected cells *via* ribosomal leaky-scanning [13] (Fig 1). P1-P5 have various functions in RABV genome transcription and replication, and in antagonism of the host cell interferon (IFN)-dependent anti-viral immune response [14–21]. P1, the most abundant isoform, acts as an essential co-factor in genome transcription/replication through direct interaction with the viral polymerase L-protein (dependent on residues 1–19 of the P1 N-terminal region (NTR)) and with the negative-sense RNA genome, through interaction with genome-associated nucleoprotein (N-protein) by the P-protein C-terminal domain (P-CTD, residues 174–297) [17,18,22,23] (Fig 1).

The shorter isoforms (P2-P5) lack the L-binding region (Fig 1) indicating that they are expressed for alternative functions, including in IFN-antagonism. All isoforms contain a site within the common P-CTD that can bind to the IFN-activated nucleocytoplasmic transcription factors signal transducers and activators of transcription (STAT) 1, 2, and 3 (Fig 1) so that different isoforms can antagonize essential functions of STATs in IFN signaling through interactions formed in the cytoplasm and in the nucleus [10,11,20,21,26,32,33]. Formation of inhibitory nuclear and cytoplasmic P-protein-STAT complexes is dependent on the differing nucleocytoplasmic localization of the isoforms, whereby P1 and P2 localize predominantly to the cytoplasm while P3-P5 are able to accumulate in the nucleus [9,13,27], due to several distinct nuclear trafficking signals. In the current model, a strong NES (N-NES) in the N-terminal region of P1 and P2 causes mostly cytoplasmic localization of these isoforms at steady state [27,28]. Transcription of P3 results in truncation and inactivation of the N-NES, and also activates an IMP-binding NLS (N-NLS) in the NTR, resulting in strong nuclear accumulation [9,27]. This is modulated by a second NES (C-NES) in the P-CTD [28]. Further truncation to generate P4 and P5 entirely deletes the N-NES, but also removes critical residues of the N-NLS (K54 and R55) to inactivate IMP binding by this sequence. Nevertheless, these isoforms localize to the nucleus, albeit to a reduced level compared with P3 [9]. In P1 the N-NLS is also inactive due to a negative regulatory effect of residues 1–52, but P1 can accumulate in the nuclei of cells in which CRM-1-mediated nuclear export is inhibited by leptomycin-B (LMB) treatment [9,27]. LMB also effects a significant increase in nuclear localization of P-protein in infected cells, the majority of which is P1, consistent with active nucleocytoplasmic trafficking during infection [34]. Together these data suggest that P1, P2, P4 and P5 contain an additional functional NLS.

The nuclear targeting mechanism of this NLS is unresolved, but prior to identification of the N-NLS, it was demonstrated that mutation of two residues in the P-CTD (K214 and R260), that are distant from the N-NLS (Fig 1), can effect exclusion of P3 from the nucleus [27]. The P-CTD crystal structure indicated that these residues, which are separated by 44 residues in the primary sequence, are brought into close proximity within a “positive patch” on the surface of the globular domain; thus, it was proposed that they might form a conformational C-terminal NLS (C-NLS) [25,27]. However, P-CTD was not shown to bind importins or to mediate IMP-dependent nuclear import; furthermore, since the C-NES had not been identified, the potential role of active export in the effect of the mutations was not investigated. Thus, the mechanistic basis of this effect is unresolved and, following the finding that the N-NLS is the major driver of P3 import [9], the potential function of the proposed C-NLS is unclear. Here, using live-cell imaging and *in vitro* protein interaction and nuclear transport assays, we show for the first time that the P-CTD binds to the IMPα2/IMPβ1 heterodimer, ΔIBB-IMPα2, and IMPβ1 alone, and mediates IMPα2/IMPβ1-dependent nuclear import, dependent on residues K214 and R260. This reveals a new model for P-protein nuclear trafficking.

## Materials and Methods

### Constructs

Constructs for mammalian cell expression of challenge virus standard 11 (CVS11)-strain RABV P-proteins/derivatives fused to the C-terminus of GFP are described elsewhere [9,28], or were generated by PCR amplification and cloning in-frame C-terminal to GFP in the pEGFP-C1 vector, as previously described [20,28]. For bacterial expression and purification, cDNA encoding the P-CTD was inserted into the pDEST-GFP-RFB vector by Gateway^™^ cloning (Invitrogen) in-frame C-terminal to His_6_-tagged GFP [9]. Mutation of K214 and R260 to alanine (K214/R260-A) in P-protein cDNA was performed by PCR as described previously [9,19].

### Cell culture, transfection, and drug treatments

COS-7, HEK293T, SH-SY5Y and HTC cells were cultured in DMEM with 10% FCS (37°C, 5% CO_2_). HeLa and T98G cell lines were cultured in EMEM with 10% FCS (37°C, 5% CO_2_). NA cells were cultured in RPMI with 10% FCS (37°C, 5% CO_2_). For analysis by confocal laser scanning microscopy (CLSM), cells were grown on coverslips to 80–90% confluence before transfection using FuGene HD reagent (Promega) and imaging 20 h later. For inhibition of CRM1-mediated nuclear export, cells were treated with 2.8 ng/ml leptomycin-B (LMB, a gift from M. Yoshida, RIKEN, Japan) for 3 h prior to analysis.

### CLSM and image analysis

Cell imaging used an Olympus FV1000 or Leica SP5 confocal laser scanning microscope with 60x oil immersion objective (NA 1.4), and heated chamber (37°C) for live-cell analysis. Image J software was used to analyze CLSM images to determine the ratio of nuclear to cytoplasmic fluorescence (F_n/c_), corrected for background fluorescence, for individual cells as previously described [9,10,19,20,35]. The mean F_n/c_ was then calculated for ≥30 cells (biological replicates) for each transfection/condition in each assay. Statistical analysis (Student’s *t*-test) was performed using GraphPad Prism software.

### Protein purification

*E*. *coli* (M15 pRep4 strain) was transformed with constructs encoding His_6_-GFP-fused T-ag NLS, P-CTD, P-CTD-K214/R260-A or TRF1 NLS. Induction of expression and purification using nickel-nitrilotriacetic acid columns (Qiagen) under denaturing conditions (8 M urea) was performed as described previously [9,36,37]. Proteins were renatured on the column and eluted with 200 mM imidazole, prior to dialysis and concentration using a 30K MWCO column (Amicon). Full-length mouse IMPα2, mouse IMPα2 lacking the autoinhibitory IMPβ1-binding domain (ΔIBB-IMPα2), and full-length mouse IMPβ1 fused to GST in pGEX-2T plasmids were expressed in *E*. *coli* (TG-1 strain) cells, and purified by affinity chromatography using glutathione-sepharose as previously described [6]. Purified GST-IMPα2 and GST-IMPβ1 was biotinylated using Sulfo-NHS-Biotin reagent (Pierce) [38].

### *In Vitro* Nuclear Transport Assay

To assess the nuclear import of purified GFP-fused proteins, the plasma membrane of rat HTC cells grown on coverslips was mechanically perforated using tissue paper, as previously described [39–41]. The perforated cells were incubated with 10 μM GFP-fusion protein, 0.5 mg/ml 70-kDa Texas red-labeled dextran (TR70, to confirm successful permeabilization of the cell membrane and maintenance of integrity of the nuclear envelope), 45 μg/μl rabbit reticulocyte lysate (Promega, to provide exogenous cytosol), and an ATP-regenerating system (0.125 g/ml creatine phosphokinase, 30 mM phosphocreatine, and 2 mM ATP). GFP protein localization was analyzed over time by CLSM, with image analysis performed using Image J, as described above. In some assays, reticulocyte lysate was pre-incubated with antibodies specific for IMPβ1 or IMPα2 (BD Transduction Labs) for 15 min at room temperature to inhibit IMP activity, as previously described [39,40].

### Native polyacrylamide gel electrophoresis (PAGE)

Purified GFP-fused proteins (2 μM) were incubated with or without 10 μM purified recombinant ΔIBB-IMPα2, IMPβ1, or pre-dimerized IMPα2/β1 heterodimer for 15 min at room temperature before electrophoresis using a pre-cast native polyacrylamide (10%) gel (Bio-Rad) in 1x TBE buffer. Migration of GFP-fused proteins was visualized using a Typhoon Trio^™^ fluoroimager (GE Healthcare LifeSciences), and gel images were processed using Image J software as described previously [9,42].

### Amplified luminescent proximity homogenous assay screen (AlphaScreen)

Purified His_6_-tagged GFP-P-CTD proteins (30 nM final concentration) were incubated with 0–60 nM biotinylated IMPβ1 or IMPβ1 heterodimerized with biotinylated IMPα2 (IMPα2/β1) for 30 min at room temperature in a 384-well plate (PerkinElmer Life Sciences). 2 μl streptavidin-coated acceptor beads (diluted 1:20 in PBS with 5% BSA) were then added to each well. After a further 90 min at room temperature, 1 μl nickel chelate-coated donor beads (diluted 1:10 in PBS) was added, followed by incubation for a further 2 h at room temperature. AlphaScreen counts were then measured using a Fusion-α^™^ plate reader (PerkinElmer Life Sciences) [38]. Curves were plotted for averaged triplicate values using GraphPad Prism software.

### Co-immunoprecipitation and Western analysis

HEK293T cells transfected to express GFP-fused proteins were lysed for immunoprecipitation using GFP-Trap beads (Chromotek) as previously described [10]. Immunoprecipitate (IP) and whole cell lysate (WCL) samples were separated by SDS-PAGE before analysis by Western blotting using antibodies against IMPα2 (BD Transduction Labs) and GFP (Abcam).

## Results

### Mutation of K214 and R260 inhibits P3 nuclear localization by a mechanism involving active nuclear export and impaired nuclear import

It was previously reported that mutation of K214 and R260 of the P-CTD to alanine (K214/R260-A) results in predominantly cytoplasmic localization of P3, leading to the proposal that P-CTD might contain a NLS (C-NLS) [27]. Subsequent studies, however, indicated that the distinct N-NLS is the principal signal in P3 nuclear localization [9]. It was also recently shown that the CTD also contains a NES (C-NES) that is partially buried within the globular CTD core (Fig 1), with another potential NES localized within residues 53–174 [9,28]. We therefore considered it possible that nuclear exclusion of K214/R260-A-mutated P3 involves NES activity, rather than being solely due to inhibition of the proposed NLS.

**Fig 2:**
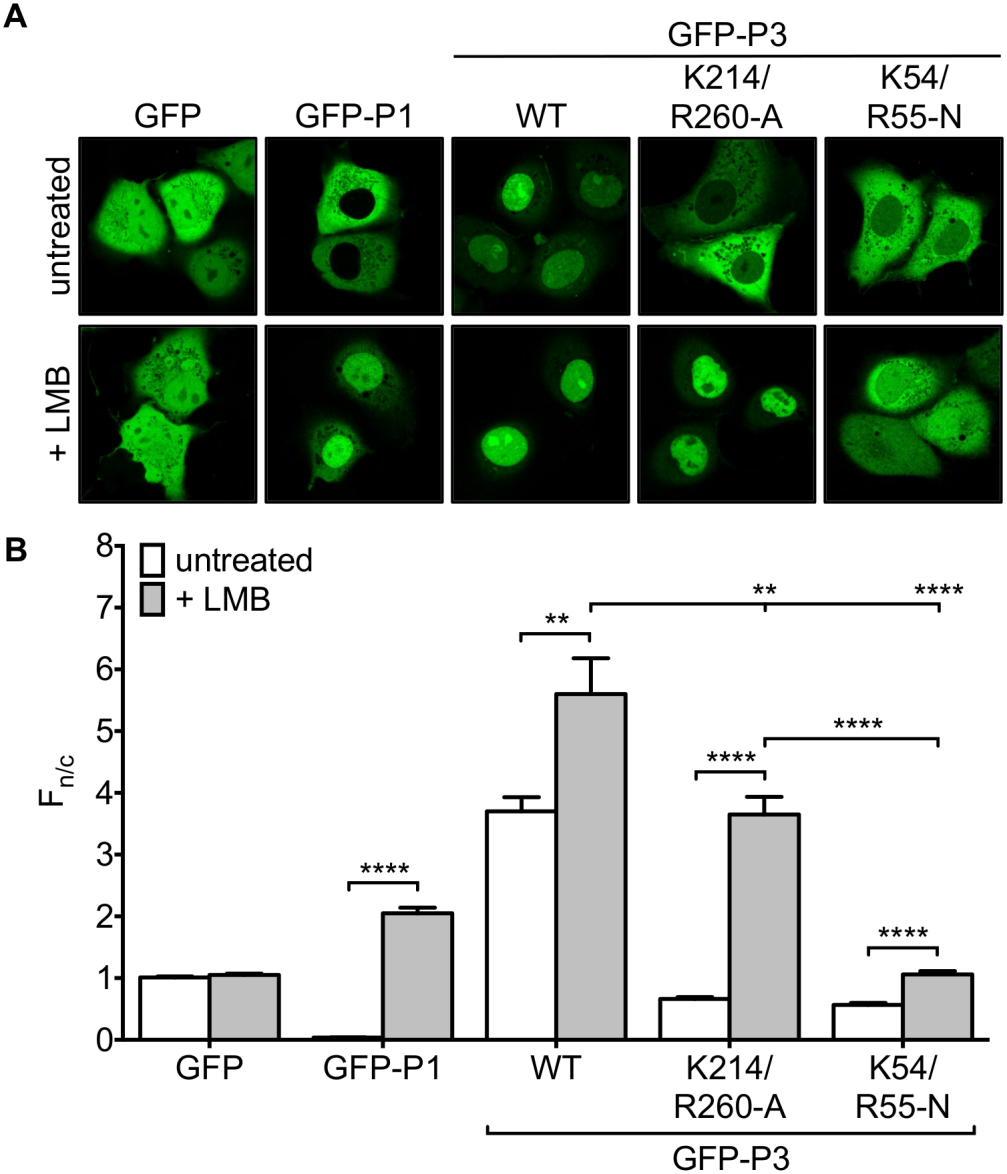
Mutation of K214/R260 affects nuclear localization of P3 through a mechanism involving active nuclear export and impaired nuclear import. (A) COS-7 cells transfected to express the indicated proteins were treated with or without LMB (2.8 ng/ml, 3 h) prior to analysis of living cells by CLSM. (B) Images such as those shown in A were analyzed to determine the ratio of nuclear to cytoplasmic fluorescence (F_n/c_) corrected for background fluorescence for individual cells as previously described [9,28]; the histogram shows the mean F_n/c_ ± S.E.M., calculated for n ≥ 30 cells for each transfection/condition. Data are representative of three separate assays. Statistical analysis used Students t-test (**, p ≤ 0.01; ****, p < 0.0001).

To examine this, we expressed GFP fused to wildtype (wt) P3, P3 containing K214/R260-A (P3-K214/R260-A), or P3 containing mutations K54-N and R55-N, that specifically inactivate the N-NLS (P3-K54/R55-N), in COS-7 cells. Analysis of live cells by CLSM (Fig 2) indicated that, consistent with previous data for GFP-fused and non-fused P-proteins [9], GFP-P3 clearly accumulates in the nucleus compared with GFP alone. As expected [9], the nuclear accumulation of GFP-P3 was strongly inhibited by K54/R55-N mutation. Importantly, mutation of K214 and R260 also strongly impaired P3 nuclear accumulation resulting in apparent exclusion from the nucleus, even though the N-NLS is intact.

To determine the potential role of NES activity in the observed effects, we analyzed cells following treatment with the CRM-1 inhibitor LMB. As expected, LMB substantially increased nuclear localization of P1, due to inhibition of CRM-1 interaction with the strong N-NES that mediates P1 nuclear exclusion, but had no effect on GFP, which lacks NES activity [27]. Consistent with previous observations, LMB treatment clearly increased the nuclear localization of GFP-P3, confirming that NES activity is important in P3 trafficking [9,27]. However, LMB had only a minor effect on the localization of GFP-P3-K54/R55-N, consistent with the fact that these mutations principally affect nuclear import by the N-NLS [9], such that GFP-P3 cannot efficiently accumulate in the nucleus even when nuclear export is disabled. In contrast, LMB treatment substantially increased nuclear localization of GFP-P3-K214/R260-A. Similar results were obtained using T98G, SH-SY5Y and HeLa cells (S1 Fig and data not shown). Thus, it appeared that K214/R260-A mutation either enhances NES activity and/or changes the relative activity of the NES and the putative C-NLS by inhibiting the function of the latter, resulting in higher net nuclear export activity, that counteracts the activity of the N-NLS. Nevertheless the N-NLS remains functional so that GFP-P3-K214/R260-A can accumulate strongly in the nucleus when nuclear export is inhibited (Fig 2).

To quantitate the effects of the mutations on P3 nuclear localization, we determined the ratio of nuclear to cytoplasmic fluorescence (F_n/c_) for each GFP-fusion protein (Fig 2B). This confirmed that GFP-P3 is significantly more nuclear than GFP, with K54/R55-N or K214/R260-A mutation resulting in a significant reduction in nuclear localization, reaching an F_n/c_ of <1, consistent with a degree of exclusion from the nucleus. As expected, LMB treatment substantially increased the F_n/c_ of GFP-P3-K214/R260-A, but had only a minor effect on GFP-P3-K54/R55-N. However, we also consistently observed that GFP-P3-K214/R260-A could not fully recapitulate the nuclear accumulation observed for GFP-P3 wt in LMB-treated cells, indicated by a significantly (p < 0.01) lower F_n/c_. Similar results were observed in all cell types tested (S1 Fig). The data thus confirmed that the N-NLS is a major driver of P3 nuclear import, but also indicated that mutations to the P-CTD can inhibit P3 nuclear import, suggesting that the P-CTD does contain NLS activity that contributes to the nuclear localization of P3.

### K214/R260 mutation prevents nuclear accumulation of P1

**Fig 3:**
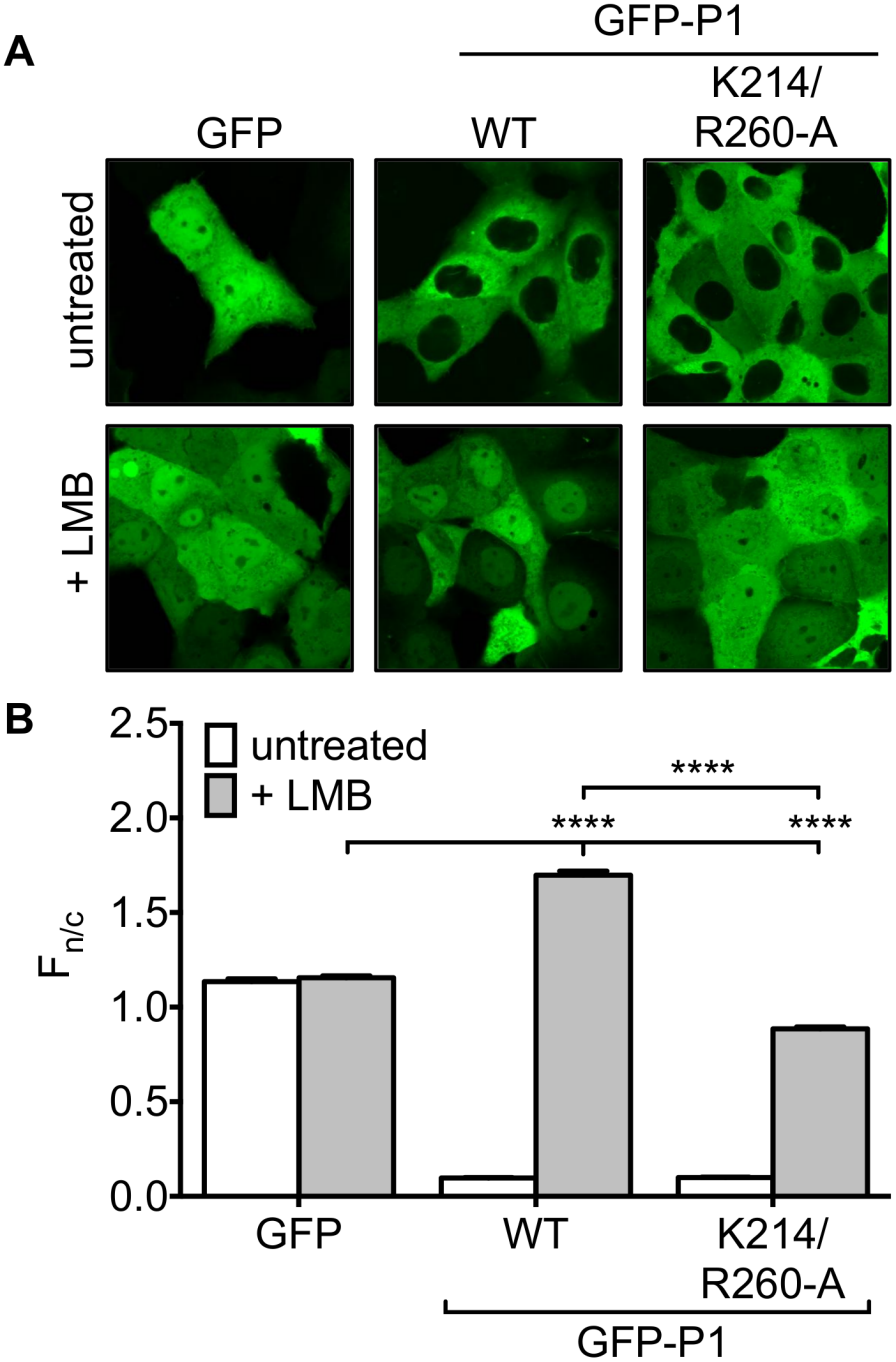
Mutation of K214/R260 prevents P1 nuclear accumulation. (A) COS-7 cells transfected to express the indicated proteins were treated without or with LMB (2.8 ng/ml, 3h) before imaging of living cells by CLSM and (B) determination of F_n/c_ as described in the legend to Fig 2 (mean ± S.E.M., n ≥ 50 cells). Data are representative of three separate assays; statistical analysis used Student’s t-test: **** p < 0.0001)

Since the N-NLS is inactive in P-protein isoforms other than P3 [9], we hypothesized that the C-NLS might play an important role in the nuclear import of P1. CLSM analysis of COS-7 cells expressing GFP-fused wt P1 or GFP-P1-K214/R260-A indicated that, consistent with previous analyses of GFP-tagged and non-tagged P1 [9], these proteins are strongly excluded from the nucleus compared to GFP, as expected due to the presence of the N-NES (Fig 3). Determination of the F_n/c_ confirmed that the nucleocytoplasmic localization did not differ between wt and mutated P1. In cells treated with LMB, GFP-P1 showed significant (p < 0.0001) accumulation in the nucleus compared with GFP. However, while the F_n/c_ of GFP-P1 K214/R260-A was increased following LMB treatment, it remained significantly (p < 0.0001) lower than the F_n/c_ for GFP, suggesting that P1 contains NLS activity in the CTD (Fig 3), important to its nucleocytoplasmic trafficking.

### P-CTD contains an IMP-binding NLS that is dependent on K214/R260

**Fig 4:**
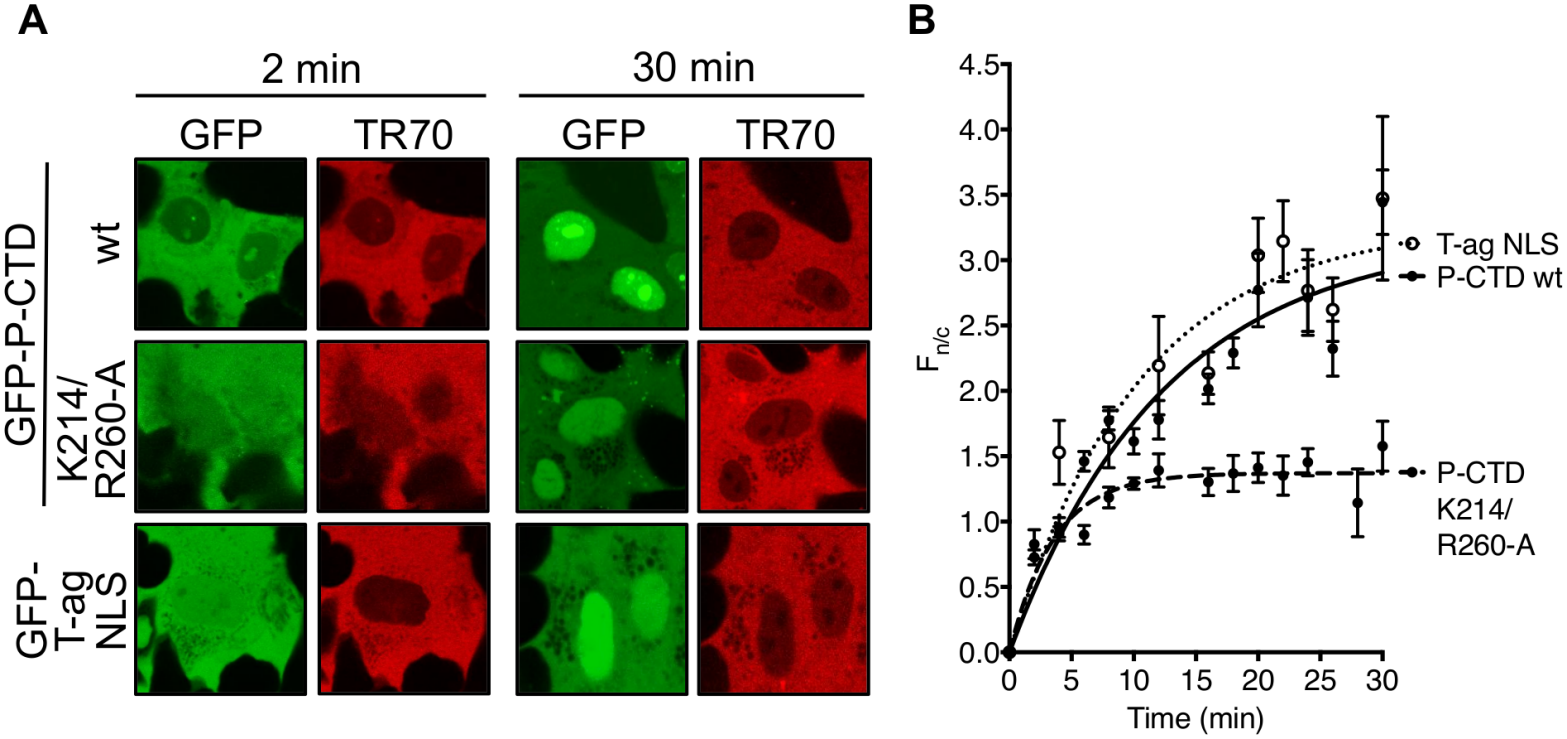
P-CTD mediates nuclear import of GFP that is inhibited by mutation of K214/R260. (A) Purified recombinant GFP-T-ag NLS, GFP-P-CTD, and GFP-P-CTD-K214/R260-A protein was added to mechanically perforated HTC cells together with exogenous cytosol (rabbit reticulocyte lysate) and an ATP-regeneration solution to reconstitute nuclear import, and TR70 to monitor nuclear envelope integrity [39,40]. Nuclear localization of the GFP-fused proteins was then monitored over 30 min by CLSM (representative images at 2 and 30 min are shown). (B) Images such as those shown in A were analyzed to determine the F_n/c_ as described in the legend to Fig 2 (mean ± S.E.M., n ≥ 3 nuclei at each timepoint). Data are from a single assay representative of two separate assays. Curve fitting used GraphPad Prism 6 (one-phase association).

To directly determine whether the P-CTD contains NLS activity, we expressed and purified recombinant His_6_-GFP-fused wt P-CTD and P-CTD-K214/R260-A. The capacity of P-CTD to mediate nuclear import was then examined using an *in vitro* nuclear import assay wherein nuclear accumulation of GFP-tagged protein added to mechanically perforated HTC cells is monitored over time by CLSM [6,39–41,43] (Fig 4A). All samples included Texas red-labeled 70kDa dextran (TR70) to monitor the integrity of the nuclear envelope based on exclusion from the nuclear compartment [39,40]; a well-defined IMPα/β recognized cargo, GFP-tagged SV40 T-ag NLS (GFP-T-ag NLS) [37,40], was used as a positive control.

**Fig 5:**
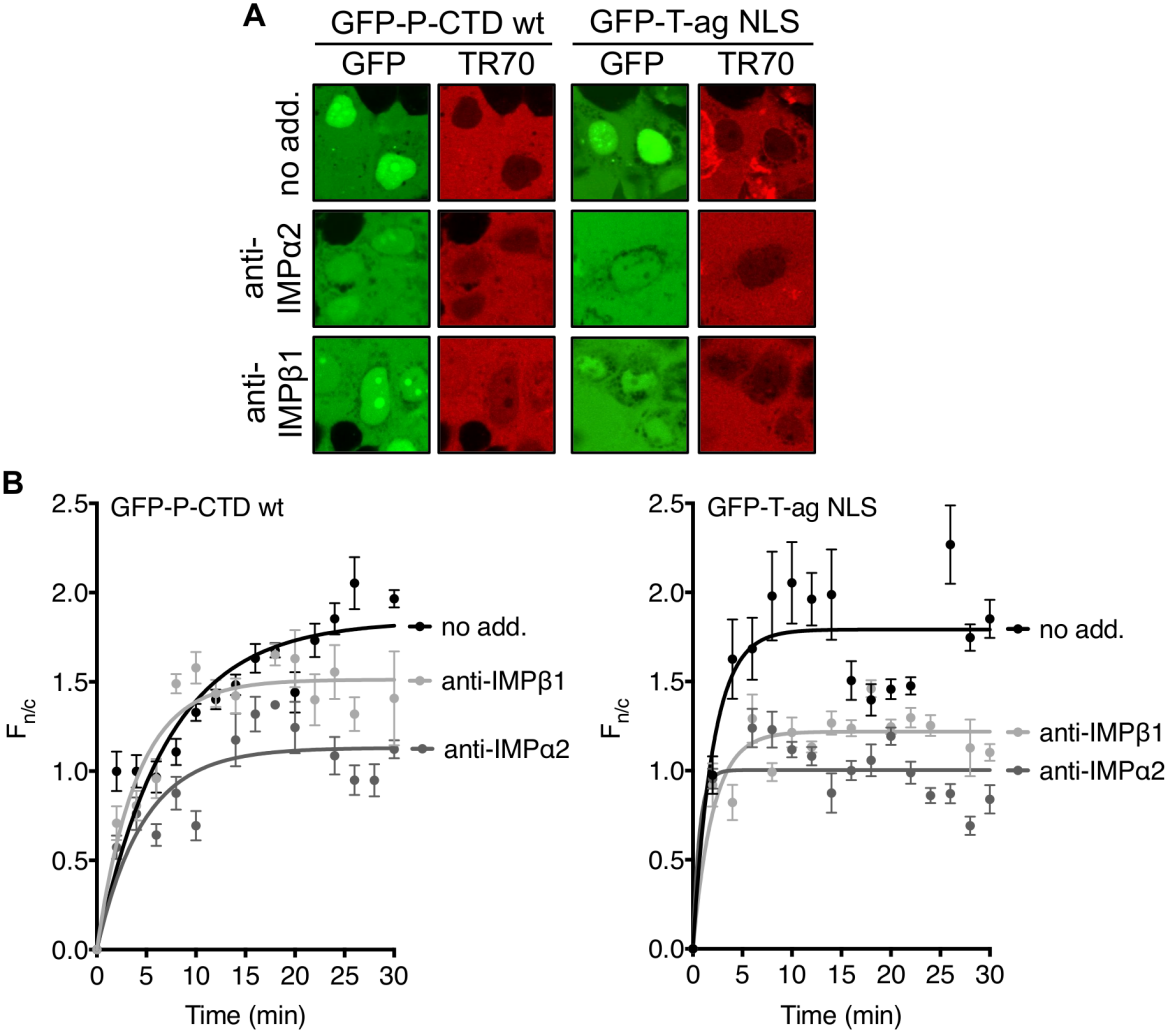
P-CTD mediates nuclear import of GFP dependent on IMPα2 and IMPβ1. Nuclear import of purified GFP-P-CTD and GFP-T-ag NLS was analyzed as described in the legend to Fig 4 except that exogenous cytosol was pre-incubated (15 min) with the indicated antibodies. (A) CLSM images following 30 min incubation of GFP-P-CTD and GFP-T-ag NLS in the presence of the indicated antibody are shown. (B) Images such as those in A were analyzed to determine the F_n/c_ as described in the legend to Fig 2 (mean ± S.E.M., n ≥ 3 nuclei for each time point; data are from a single assay typical of three separate assays). Curve fitting used GraphPad Prism 6 (one-phase association).

CLSM analysis indicated that GFP-P-CTD accumulated in the nucleus to an extent comparable to the GFP-T-ag NLS control, whereas the accumulation of GFP-P-CTD-K214/R260-A was greatly reduced, consistent with impaired NLS activity (Fig 4B). To assess the role of IMPα2 and β1, we examined the effect of inhibitory antibodies, finding that both anti-IMPα2 and anti-IMPβ1 strongly inhibited nuclear accumulation of the IMPα/β-dependent T-ag NLS, as expected [40], as well as that of GFP-P-CTD (Fig 5). Thus, it appears that the P-CTD can confer IMPα2/β1-dependent nuclear import to GFP, consistent with NLS functionality.

### P-CTD binds directly to IMPs, dependent on K214/R260

**Fig 6:**
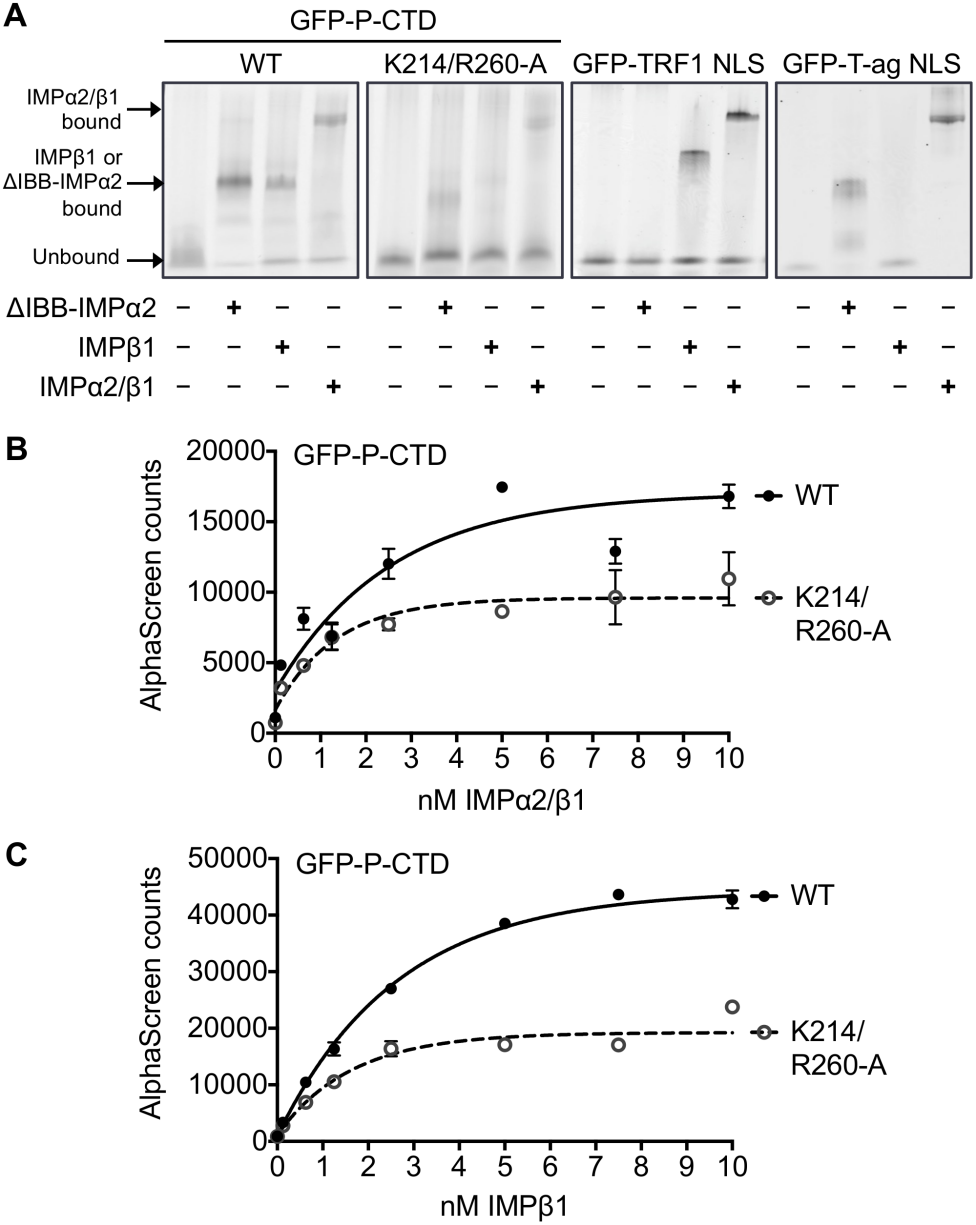
P-CTD interacts with IMPα2, IMPβ1 and IMPα2/β1 dependent upon K214/R260. (A) 2 μM GFP-fused wt P-CTD, P-CTD-K214/R260-A, TRF1 or T-ag NLS was incubated with 10 μM GST-fused mouse ΔIBB-IMPα2, IMPβ1 or IMPα2/β1 before native gel electrophoresis and fluoroimaging as previously described [9,39,42]. Binding to IMPs is indicated by altered mobility of the GFP-fused proteins compared to that of no IMP controls (different complexes are indicated by arrows). Data is from a single assay representative of two independent assays. (B, C) 30 nM His_6_-tagged GFP-P-CTD wt or GFP-P-CTD-K214/R260-A was incubated with increasing concentrations of (B) biotinylated-IMPα2/β1 or (C) biotinylated-IMPβ1 prior to AlphaScreen analysis. Assays were performed in triplicate; graphs show mean AlphaScreen counts ± S.E.M. (n = 3) from a single typical experiment, representative of four independent assays.

To examine whether the P-CTD interacts directly with IMPs, we used a native gel mobility shift assay as described [42]. The best understood NLS-dependent nuclear import pathway involves NLS recognition by the IMPα/β1 heterodimer, where the cargo interacts directly with IMPα, which in turn is bound to IMPβ1 through IMPα’s IMPβ binding (IBB) domain. In the absence of IMPβ1, IMPα conventionally binds NLS-containing cargoes only weakly, but strong binding can be observed for the “ΔIBB” form of IMPα that lacks the autoinhibitory IBB [44]. GFP-fused recombinant P-CTD, P-CTD-K214/R260-A, TRF1 NLS (a known IMPβ1 binding protein) [36,37], or T-ag NLS was incubated with GST-fused ΔIBB-IMPα2, IMPβ1 alone or IMPα2/IMPβ1 heterodimer, before analysis by native gel electrophoresis and fluoroimaging. Consistent with previous data [42], ΔIBB-IMPα2 and the IMPα2/β1 dimer, but not IMPβ1 alone, induced a clear shift in mobility of GFP-T-ag NLS, indicative of direct interaction with the heterodimer *via* IMPα2. IMPβ1 and IMPα2/β1, but not ΔIBB-IMPα2, induced a shift in mobility for GFP-TRF1 NLS, consistent with the reported IMPβ1-dependent mechanism [36] (Fig 6A). Analysis of GFP-P-CTD clearly indicated binding to ΔIBB-IMPα2, IMPβ1 and the IMPα2/β1 dimer. This suggests that the CTD can not only interact with IMPα2 and the IMPα2/β1 dimer, indicative of an IMPα/β heterodimer-dependent mechanism, but also with IMPβ1 alone. Importantly the observed shift in mobility for all IMPs/complexes was strongly impaired for GFP-P-CTD-K214/R260-A (Fig 6A). Similar results were derived in AlphaScreen assays, which were used to estimate the affinity of the binding interaction of GFP-P-CTD with either the IMPα2/β1 heterodimer (Fig 6B) or IMPβ1 alone (Fig 6C); this indicated apparent dissociation constants (*K*_*d*_s) of 1.7 ± 0.6 and 1.5 ± 0.4 nM, respectively. Importantly, both IMPα2/β1 and IMPβ1 showed a c. 50% reduction in maximal binding (B_max_) by GFP-P-CTD-K214/R260-A compared to wt GFP-P-CTD (B_max_ for IMPα2/β1 = 9599 ± 546.6 (GFP-P-CTD-K214/R260-A) and 17041 ± 971.6 (GFP-P-CTD wt); for IMPβ1 = 19242 ± 633.2 (GFP-P-CTD-K214/R260-A) and 44383 ± 624.9 (GFP-P-CTD wt)) (Fig 6B and 6C). Thus, it appears that the P-CTD contains an NLS that can be recognized with high (low nM) binding affinity by either IMPα/β or IMPβ1, dependent on K214 and R260.

**Fig 7:**
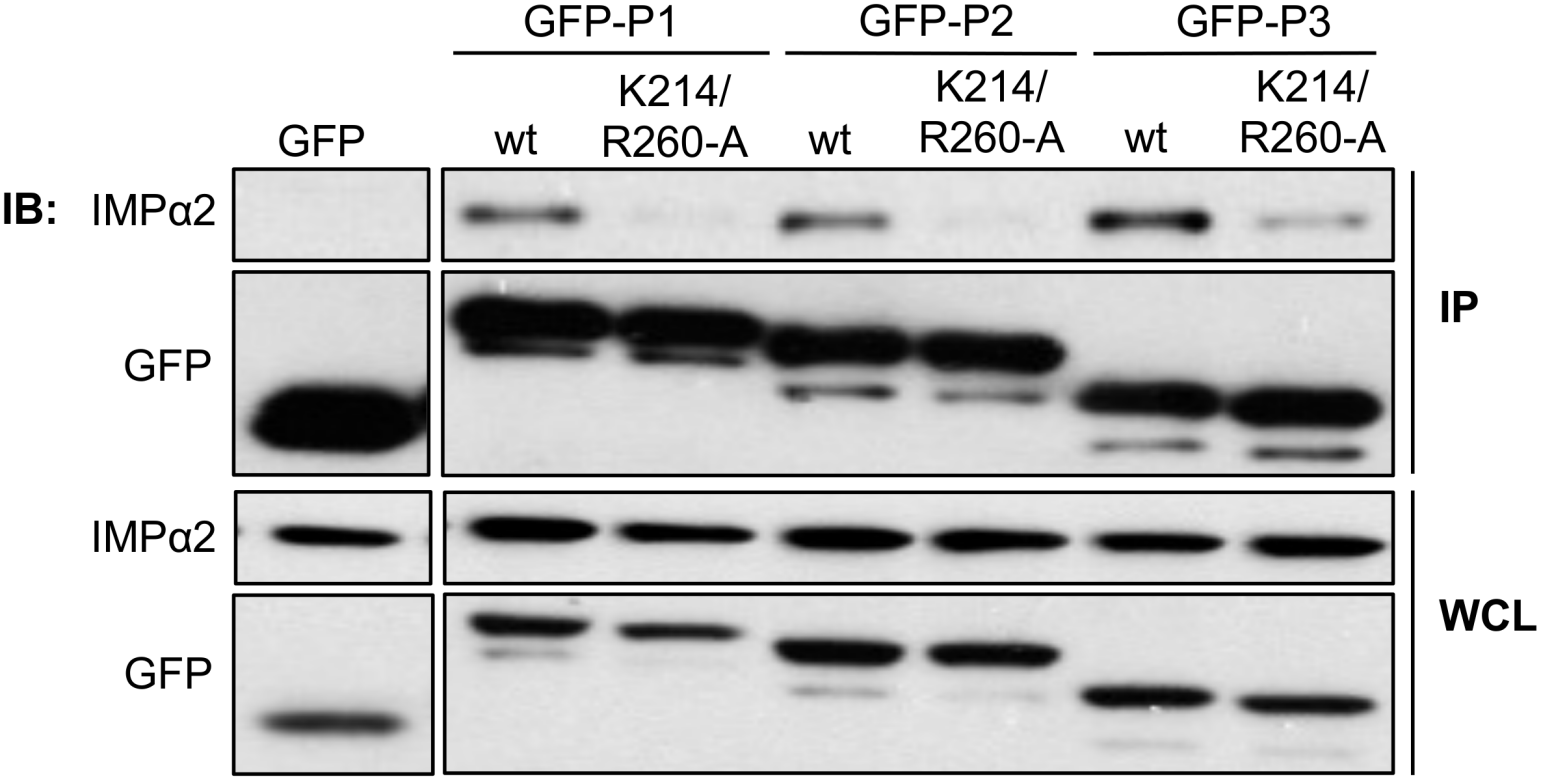
IMPα2 co-precipitates with RABV P-proteins from mammalian cells, dependent on K214/R260. HEK293T cells expressing the indicated GFP-fused proteins were subjected to immunoprecipitation using GFP-Trap before analysis of immunoprecipitate (IP) and whole cell lysate (WCL) by immunoblotting (IB) for IMPα2 and GFP. Data is from a single assay, representative of three independent experiments.

To confirm that P-CTD interacts with IMPs in mammalian cells, we expressed GFP-fused P1, P2, or P3 (wt and K214/R260-A-mutated derivatives) in HEK293T cells before immunoprecipitation and Western analysis using anti-IMPα2 antibody (Fig 7). IMPα2 co-precipitated with all wt P-proteins, but not with GFP alone. Importantly, the K214/R260-A mutation clearly reduced co-precipitation of IMPα2 in all cases. Similar results were observed in co-immunoprecipitation assays using mouse neuroblastoma NA cells expressing GFP-P-CTD wt or GFP-P-CTD K214/R260-A (S2 Fig). Of note, we consistently observed that although the amount of IMPα2 co-precipitated by GFP-P3-K214/R260-A was lower than that precipitated by GFP-P3 wt, it remained greater than that precipitated by other mutated isoforms, consistent with the presence of the functional N-NLS in P3, that is inactive or deleted from other isoforms [9] (Fig 7).

Together, these data indicate that the nuclear import of P3 is driven by the C-NLS together with the N-NLS, and that the C-NLS is the main driver of IMP-interaction and IMP-dependent nuclear import of P-protein isoforms lacking the N-NLS.

## Discussion

Here, we have shown for the first time that the P-CTD contains a genuine IMP-binding NLS that can confer high affinity interaction with either the IMPα2/β1 heterodimer or IMPβ1 alone, and mediates IMP-dependent nuclear import. Importantly, the finding that nuclear trafficking of the major P-protein isoform P1 is dependent on the C-NLS, which is physically and functionally distinct from the previously determined N-NLS of P3, provides the first mechanistic insights into how P1 and other isoforms lacking N-NLS function can be imported to the nucleus.

RABV is a negative-sense single stranded RNA virus, which replicates its genome entirely within the cytoplasm. Nevertheless, our data indicate roles for precisely regulated nuclear import of the P-protein within the host cell. Previous studies have identified a complex array of interaction sequences that define the subcellular localization of P-protein isoforms, including two clearly defined NESs [27,28], two sequences that interact with components of the cytoskeleton [20,45], and a single IMP-binding N-NLS [9]. P-protein employs highly efficient mechanisms to regulate these signals, including the expression of overlapping N-NES and N-NLS sequences, which enables co-regulation to effect strong nuclear export of P1 (in which the N-NES is active and the N-NLS inactive), or strong nuclear localization of P3 (in which truncation of residues 1–52 inactivates the N-NES and concomitantly activates the N-NLS) [9]. Our demonstration that the predicted C-NLS comprises a functional IMP-binding sequence provides new insights into the regulation of P-protein nucleocytoplasmic localization, wherein the co-localization of the IMP-binding sequence and the C-NES in the globular P-CTD is likely to effect efficient co-regulation analogous to that of the N-NLS/N-NES module (Fig 1).

It was previously shown that K214 and R260 are aligned to form part of a positive patch on the surface of the P-CTD, dependent on its 3-dimensional fold [25,27], and also flank the C-NES [28], which is largely buried in the core of the P-CTD crystal structure [25]. We have suggested that this might enable co-regulation of the predicted C-NLS and C-NES sequences through the formation of distinct conformations in which either the C-NLS/positive patch is functional and the C-NES buried, or the C-NES is exposed resulting in disassembly of the C-NLS [28]. It seems likely that the conformation of the CTD is dynamic, enabling switching between “import” and “export” conformations with mechanisms favouring one or other conformation enable rapid changes in localisation. Our current data indicate that when the IMP binding C-NLS is disabled by mutation, a strong relocalization out of the nucleus is observed for P3 due to a relative increase in NES activity, such that nuclear export activity can predominate over the strong N-NLS, consistent with an important role for the C-NLS/C-NES in regulating localization. Notably, the finding that the CTD is recognized by ΔIBB-IMPα2 and the IMPα/β heterodimer (consistent with a “classical” NLS) as well as IMPβ1 alone suggests that this region enables RABV P-protein to participate in several trafficking pathways. Since all interactions depend on K214/R260, it appears that the interactions are mediated by overlapping sites involving the positive patch, or by the same site/NLS. The capacity of this region to interact with different IMP nuclear trafficking pathways may be enabled by the fact that the C-NLS is a non-classical conformational NLS, formed dependent on the CTD domain fold rather than as a linear sequence. Given the apparently novel properties of the C-NLS compared with previously classified NLSs, it will be of interest in the future to determine the regions within IMPs that are bound by the P-CTD, and how these compare with other classes of NLS [46].

**Fig 8:**
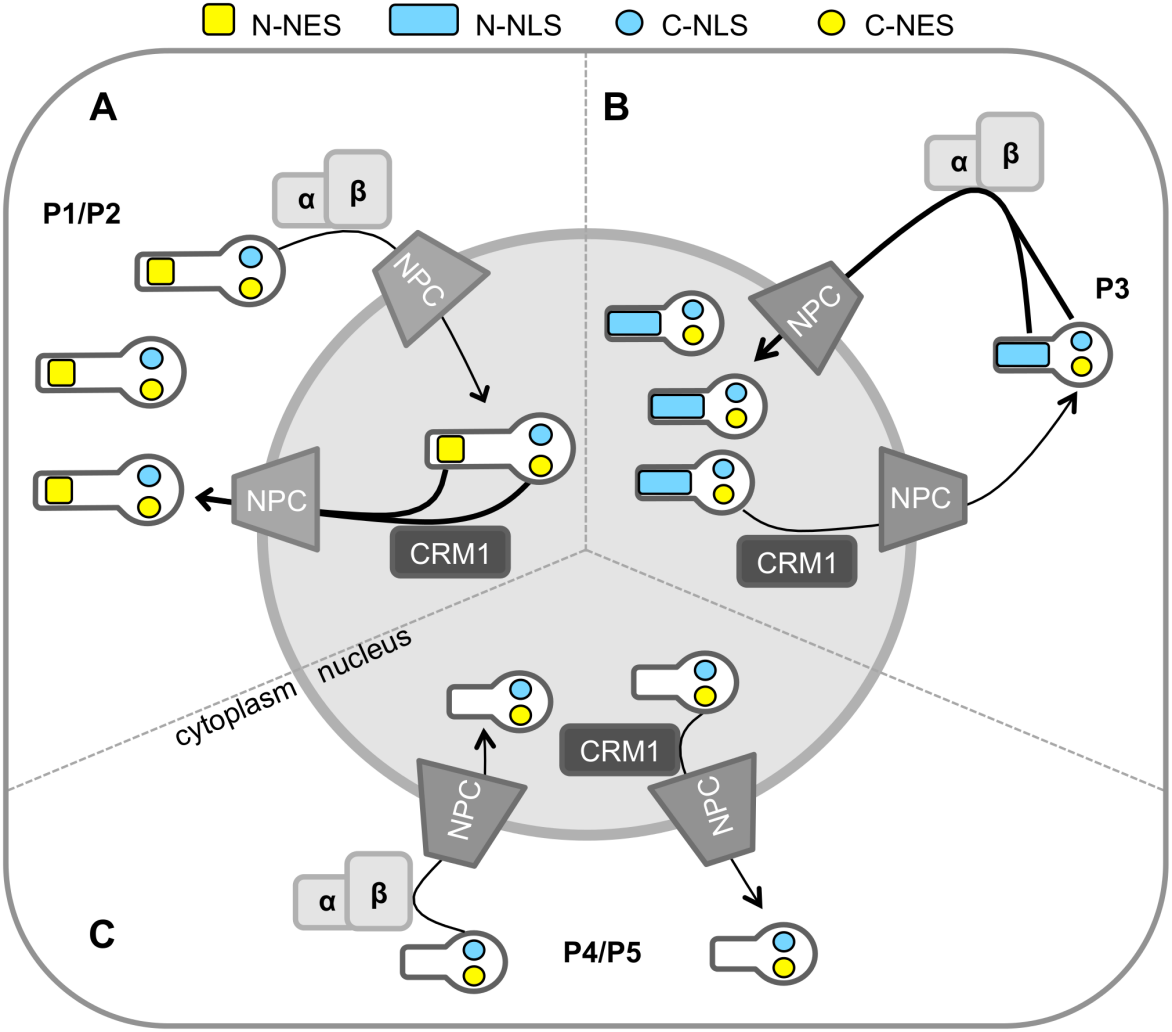
Model of rabies P protein isoform nuclear trafficking. (A) P1 and P2 localize to the cytoplasm due to the activity of the strong N-NES together with the C-NES (yellow rectangles and circles, respectively) which counters nuclear import mediated by the C-NLS alone (blue circle) [27,28]. (B) P3 accumulates within the nucleus *via* import driven by the N-NLS [9] (blue rectangle) and C-NLS. (C) Nucleocytoplasmic localization of P4-5 is diffuse, due trafficking driven by the C-NLS and C-NES only. Transport is indicated by arrows, with thicker arrows indicating the predominant direction.

Together, these data indicate that P-protein undergoes complex, highly regulated interactions with the host nuclear trafficking machinery, with localization dependent on the balance between binding to CRM1 and to IMPα/β through C- and N-terminal modules. We thus propose a refined model where C-NLS-IMP-binding plays major roles in the trafficking of P-protein isoforms (Fig 8). Specifically, P1 undergoes trafficking mediated by the IMP-binding C-NLS and the strong N-NES, resulting in largely cytoplasmic localization at steady state; P2 is likely to be regulated in similar fashion. In P3 the N-NES is inactivated and strong nuclear import is driven by the activated N-NLS together with the C-NLS, resulting in substantially greater nuclear accumulation of P3 than other isoforms. In P4/P5 the N-NLS is absent, resulting in nuclear import mediated by the C-NLS alone, with dynamic switching of the CTD between import and export conformations resulting in a relatively diffuse distribution.

A number of additional mechanisms are implicated in regulating P-protein localization. PKC-mediated phosphorylation of the P-CTD proximal to the positive patch decreases nuclear accumulation [28], and our findings indicate that this is likely to involve effects on IMPα/β interaction analogous to other viral NLSs such as those in Epstein-Barr virus nuclear antigen 1 protein and the bovine papillomavirus type 1 E1 protein [47,48]. Oligomerization of P-protein regulates nuclear localization by effecting interaction with microtubules to sequester P to the cytoskeleton [20]. Interactions with viral L- and N-proteins [17,18,24,30,31] are also likely to physically sequester P1 into replication complexes in cytoplasmic viral *Negri bodies* [49]. Since the positive patch/C-NLS is involved in binding to N-protein-genome complexes [24,50] it is likely that this interaction will additionally exclude interaction with IMPα/β, such that P1 engaged in replication would be prevented from coupling to IMP-mediated transport pathways. P-CTD also interacts with cellular proteins important in IFN-signaling, including STAT1, 2 and 3 and PML protein [11,21,26,32,51]. Notably, the predicted STAT1-binding site is separate from the positive patch within the P-CTD, suggesting that P-protein might bind to STATs and IMPs concurrently to enable roles of nuclear trafficking in IFN-antagonism [19]. Importantly, the nuclear export of P-protein has been directly implicated in IFN antagonist function *in vitro* and pathogenicity *in vivo*, indicating that nuclear trafficking is critical to infection [52], such that other trafficking sequences including C-NLS are likely to have significant roles.

In conclusion, our data show that the CTD interacts with IMPs to mediate nuclear localization of multiple P-protein isoforms, extending our understanding of the complex interface formed by P-protein with the host cell trafficking machinery. A greater knowledge of the precise sequences and mechanisms underpinning trafficking of specific P-protein isoforms is important to our understanding of rabies disease and, consequently, the identification of novel potential targets for antiviral therapeutics and vaccines for RABV, a continuing global health threat that causes over 60,000 human deaths per year [53].

## Supporting Information

S1 Fig Mutation of K214/R260 affects nuclear localization of P3 in neuronal and glial cells through a mechanism involving active nuclear export and impaired nuclear import. (A, B) T98G human glioblastoma and (C, D) SH-SY5Y human neuroblastoma cells transfected to express the indicated proteins were treated with or without LMB (2.8 ng/ml, 3 h) prior to analysis of living cells by CLSM. (B, D) Images such as those shown in A & C were analyzed to determine the ratio of nuclear to cytoplasmic fluorescence corrected for background fluorescence as previously described [9, 28] (mean F_n/c_ ± S.E.M., n ≥ 10). Statistical analysis used Students t-test (*, p<0.05; ***, p <0.001; ****, p < 0.0001).
(TIF)

S2 Fig IMPα2 co-precipitates with RABV P-proteins from neuronal cells, dependent on K214/R260. NA mouse neuroblastoma cells expressing the indicated GFP-fused proteins were subjected to immunoprecipitation using GFP-Trap before analysis of immunoprecipitate (IP) and whole cell lysate (WCL) by immunoblotting (IB) for IMPα2 and GFP.
(TIF)
