# Supplementary figures and images for: Nuclear Trafficking of the Rabies Virus Interferon Antagonist P-Protein Is Regulated by an Importin-Binding Nuclear Localization Sequence in the C-Terminal Domain
Source: PLoS One. 2016 Mar 3;11(3):e0150477. doi: 10.1371/journal.pone.0150477 (PMC4777398; doi:10.1371/journal.pone.0150477)

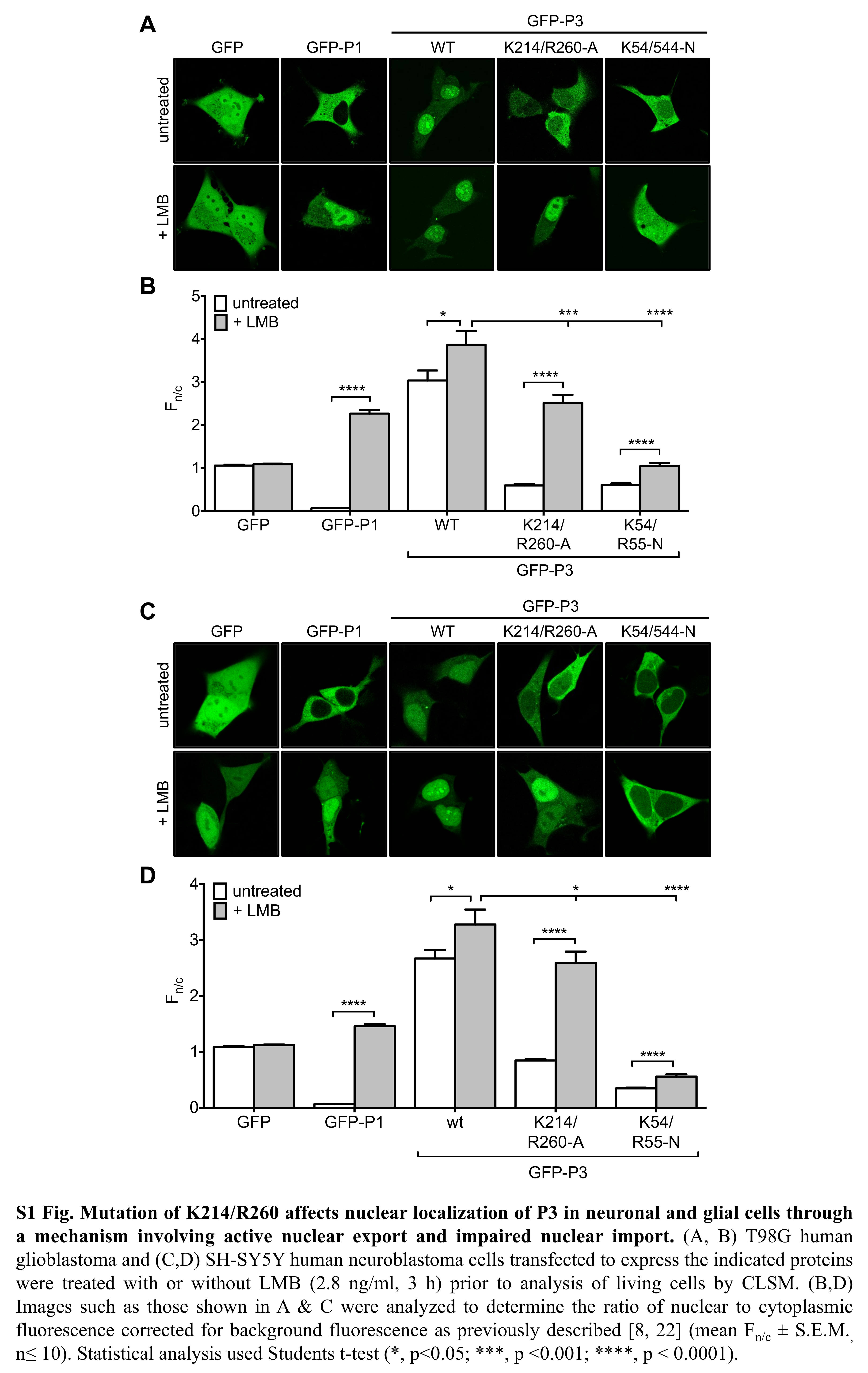

Supplement: S1 Fig — (A, B) T98G human glioblastoma and (C, D) SH-SY5Y human neuroblastoma cells transfected to express the indicated proteins were treated with or without LMB (2.8 ng/ml, 3 h) prior to analysis of living cells by CLSM. (B, D) Images such as those shown in A & C were analyzed to determine the ratio of nuclear to cytoplasmic fluorescence corrected for background fluorescence as previously described [9, 28] (mean Fn/c ± S.E.M., n ≥ 10). Statistical analysis used Students t-test (*, p<0.05; ***, p <0.001; ****, p < 0.0001). (TIF) [file pone.0150477.s001.tif]

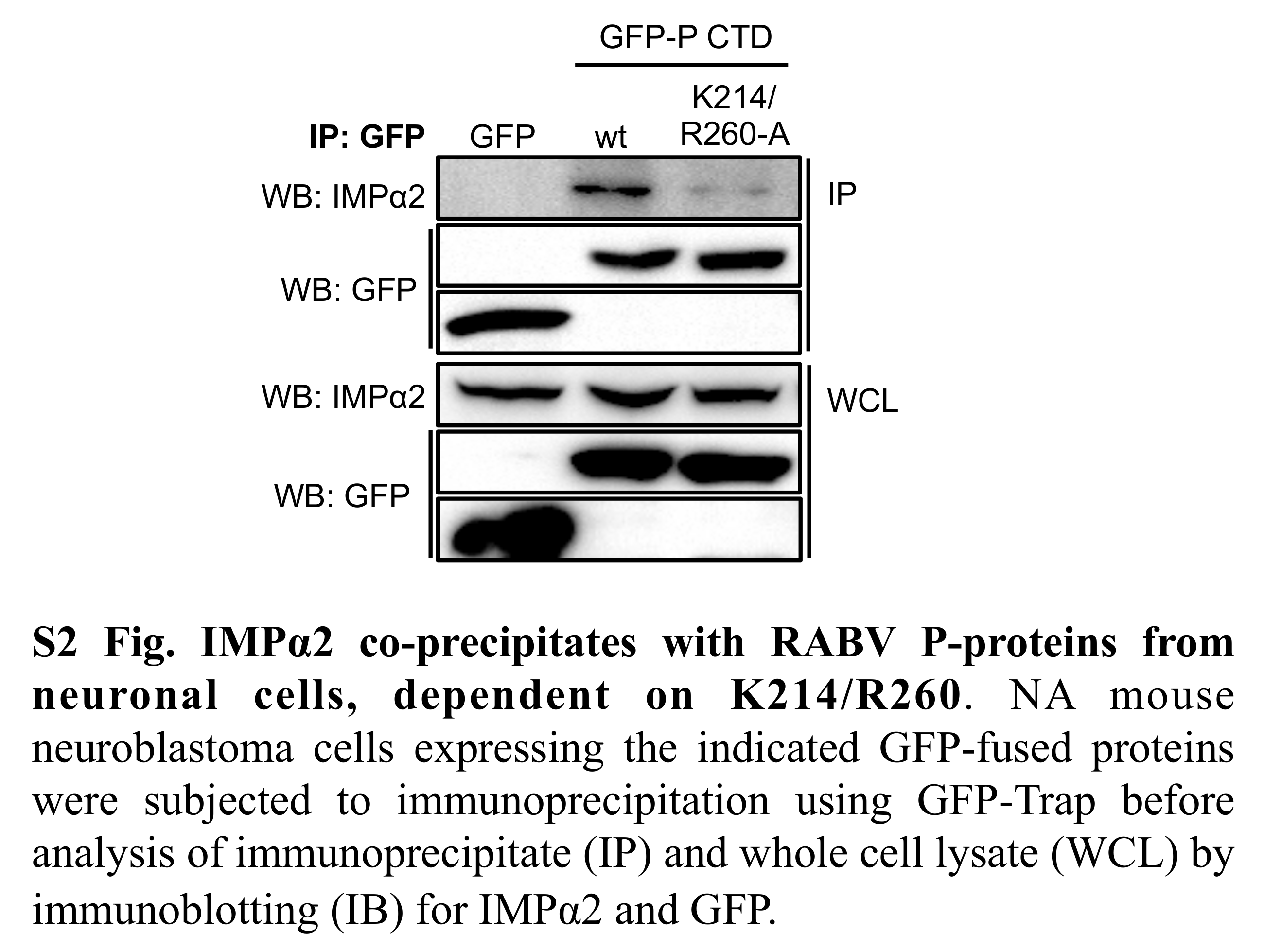

Supplement: S2 Fig — NA mouse neuroblastoma cells expressing the indicated GFP-fused proteins were subjected to immunoprecipitation using GFP-Trap before analysis of immunoprecipitate (IP) and whole cell lysate (WCL) by immunoblotting (IB) for IMPα2 and GFP. (TIF) [file pone.0150477.s002.tif]
